# The Expressions of NF−κB, COX−2, Sp1, and c−Jun in Pancreatic Ductal Adenocarcinoma and Their Associations with Patient Survival

**DOI:** 10.3390/pathophysiology30020009

**Published:** 2023-03-25

**Authors:** Kaka Renaldi, Marcellus Simadibrata, Nur Rahadiani, Diah Rini Handjari, Alida Roswita Harahap, Kuntjoro Harimurti, Nasrul Zubir, Lianda Siregar, Imelda Maria Loho, Evlina Suzanna, Bonita Prawirodihardjo, Heriawaty Hidajat, Budi Widodo, Alphania Rahniayu, Renaningtyas Tambun, Andy William, Dadang Makmun

**Affiliations:** 1 Doctoral Program in Medical Sciences, Faculty of Medicine, Universitas Indonesia, Jakarta 10430, Indonesia; 2 Division of Gastroenterology, Pancreatobiliary and Gastrointestinal Endoscopy, Faculty of Medicine, Universitas Indonesia/Dr. Cipto Mangunkusumo Hospital, Jakarta 10430, Indonesia; 3 Department of Internal Medicine, Faculty of Medicine, Universitas Indonesia/Dr. Cipto Mangunkusumo Hospital, Jakarta 10430, Indonesia; 4 Department of Anatomical Pathology, Faculty of Medicine, Universitas Indonesia/Dr. Cipto Mangunkusumo Hospital, Jakarta 10430, Indonesia; 5 Division of Gastroenterology, Department of Internal Medicine, Dr. M. Djamil Hospital, Padang 25171, Indonesia; 6 Division of Gastroenterohepatology, Dharmais National Cancer Hospital, Jakarta 11420, Indonesia; 7 Department of Anatomical Pathology, Dharmais National Cancer Hospital, Jakarta 11420, Indonesia; 8 Department of Anatomical Pathology, Fatmawati Central General Hospital, Jakarta 12430, Indonesia; 9 Department of Anatomical Pathology, Persahabatan Central General Hospital, Jakarta 13230, Indonesia; 10 Division of Gastroenterology, Department of Internal Medicine, Faculty of Medicine, Universitas Airlangga−Dr. Soetomo General Academic Hospital, Surabaya 60286, Indonesia; 11 Department of Anatomical Pathology, Faculty of Medicine, Universitas Airlangga−Dr. Soetomo General Academic Hospital, Surabaya 60286, Indonesia; 12 Department of Anatomical Pathology, St. Carolus Hospital, Jakarta 10440, Indonesia

**Keywords:** pancreatic neoplasms, nuclear factor kappa−B, cyclooxygenase−2, Sp1, c−Jun

## Abstract

Chronic inflammation is a crucial driver of carcinogenesis in pancreatic ductal adenocarcinoma (PDAC). Several studies have investigated the prognostic significance of cyclooxygenase−2 (COX−2) expression in PDAC patients, obtaining conflicting results. Nuclear factor kappa−B (NF−κB), specificity protein 1 (Sp1), and c−Jun are known as the transcription factors of the *COX2* gene. This exploratory observational study investigated the association of the NF−κB, COX−2, Sp1, and c−Jun expressions with patient survival in PDAC. We used the immunohistochemical method to detect the PDAC tissue expressions of NF−κB (RelA/p65), COX−2, Sp1, and c−Jun. The expressions of these proteins were correlated with the overall survival (OS) and other clinicopathological characteristics of PDAC patients. We obtained 53 PDAC specimens from resections and biopsies. There were significant correlations between the four proteins’ expressions in the PDAC tissues. The expression of the cytoplasmic (aHR = 0.31; 95% CI 0.11–0.90; *p* = 0.032) or nuclear NF−κB (aHR = 0.22; 95% CI 0.07–0.66; *p* = 0.007) was independently associated with a better prognosis in the PDAC patients. COX−2, Sp1, and c−Jun showed no significant association with a prognosis in the PDAC patients. The PDAC patients who expressed NF−κB had a better prognosis than the other patients, which suggests that the role of inflammation in PDAC is more complex than previously thought.

## 1. Introduction

Pancreatic cancer is the tenth most common cancer in men and the eighth most common cancer in women worldwide. It was also the seventh most common cause of cancer−related mortality in 2020, being attributed to 466,603 deaths [1]. Overall, its five−year survival rate is around 11% [2]. Its lack of effective therapies reflects the complex pathophysiology of pancreatic cancer.

Pancreatic ductal adenocarcinoma (PDAC) is the most common type of pancreatic cancer. The pathogenesis of PDAC begins with *KRAS* oncogene mutation. The *KRAS* mutation is found in 90% of patients with PDAC [3]. This mutation activates various downstream pathways, in which Phosphatidylinositol 3−kinase (PI3K)/3−phosphoinositide−dependent protein kinase−1 (Pdk1)/Akt, Raf/MEK/ERK, and the Ral guanine nucleotide exchange factor pathway are the three major pathways. The increased activation of these pathways promotes cell survival, proliferation, and invasiveness [4]. However, mutated *KRAS* alone cannot sustain oncogenic activity. Instead, a stimulus such as chronic inflammation is needed for the persistence of the *KRAS* activation [5].

Cyclooxygenase−2 (COX−2) is an enzyme that is produced in an inflammatory state. In the basal state, the *COX2* gene is only expressed at a low level in organs such as the kidneys and the central nervous system [6]. During a pro−inflammatory state, growth signals upregulate the *COX2* gene expression. The COX−2 enzyme then converts arachidonic acid into various types of prostaglandins. Prostaglandin E2 (PGE2) has been associated with oncogenic effects due to its promotion of the proliferation, migration, angiogenesis, immunosuppression, and survival of tumor cells [7]. An increased COX−2 expression has been observed in various malignancies, such as colon, breast, lung, prostate, and esophageal cancers [8]. Several studies using the tissues from PDAC patients have also demonstrated the COX−2 overexpression in the cancer cells in comparison with the adjacent non−cancerous cells from the same patients [9,10]. This increased expression is due to upregulations at the transcriptional and post−transcriptional levels [8].

Several transcription factors that regulate the *COX2* gene expression include nuclear factor kappa−B (NF−κB), specificity protein 1 (Sp1), and c−Jun [11,12]. Only a few studies have investigated their expressions in PDAC patients. NF−κB is a family of transcription factors that includes RelA (p65), RelB, c−Rel, NF−κB1 (p50/p105), and NF−κB2 (p52/p100) [13]. RelA/p65 is the most frequently studied among these four subunits. Zhang JJ et al. showed that the positive NF−κB p65 expression rate was higher in the PDAC tissues compared with the normal tissues (66.5% vs. 31.58%) [14]. Sp1 is an Sp/Krüppel−like factor (KLF) that consists of other proteins, such as Sp3 and Sp4. Sp1 is often called the basal transcription factor or the housekeeping gene, because it regulates many of the genes that are necessary for normal cellular processing, including *COX2* [15]. Hu J et al. reported that the positive Sp1 expression was higher in the PDAC tissues than in the normal tissues, although they did not report its exact percentage [16]. The protein c−Jun is one of the subunits that forms activator protein−1 (AP−1). AP−1 is a homo− or heterodimer consisting of leucine proteins from the Jun (c−Jun), Fos (c−Fos), JDP, and ATF families [11]. The c−Jun subunit of AP−1 has also demonstrated oncogenic activity [17]. Ferrara C et al. reported that the percentage of c−Jun staining ranged from 10–90% of the cells in the PDAC tissues, while only 10% of the ductal cells were stained in the normal tissues [18]. However, no studies have investigated the co−expression of the four proteins COX−2, NF−κB, Sp1, and c−Jun in PDAC tissues. 

It is important that we study these proteins, because their overexpression could potentially represent prognostic factors in cancers. Several studies have investigated the utility of COX−2 expression for predicting the patient survival in PDAC, with conflicting results [19,20,21,22]. A meta−analysis by Wang et al. reported that the PDAC patients who expressed COX−2 had worse survival than those who did not [23]. However, a more recent meta−analysis that was published by our group showed that this association was not significant when controlling for other variables [24]. Compared to those on COX−2, few studies have investigated the effects of the NF−κB, Sp1, and c−Jun expressions on PDAC patient survival [14,16,18]. Therefore, in this study, we aimed to evaluate the expressions and prognostic significance of these four proteins in PDAC patients. Our study found that NF−κB expression was associated with a better prognosis in the PDAC patients. We present the following article in accordance with the STROBE reporting checklist.

## 2. Materials and Methods

### 2.1. Study Design

We conducted an exploratory observational study that investigated the association of the NF−κB (RelA/p65), COX−2, Sp1, and c−Jun expressions with the patient survival in PDAC.

### 2.2. Location and Period of Study

We obtained the tissue samples from the PDAC patients from Cipto Mangunkusumo National General Hospital, Fatmawati Hospital, Dharmais Cancer Hospital, Persahabatan Hospital, and Dr. Soetomo Hospital, who fulfilled the study criteria. We conducted the study from April 2020 to April 2022.

### 2.3. Ethical Approval

The study was conducted in accordance with the Declaration of Helsinki (as revised in 2013). The study was approved by the Institutional Board of Fakultas Kedokteran Universitas Indonesia (No. KET−522/UN2.F1/ETIK/PPM.00.02/2020). Informed consent was obtained from the patients.

### 2.4. Patient Selection

**Figure 1:**
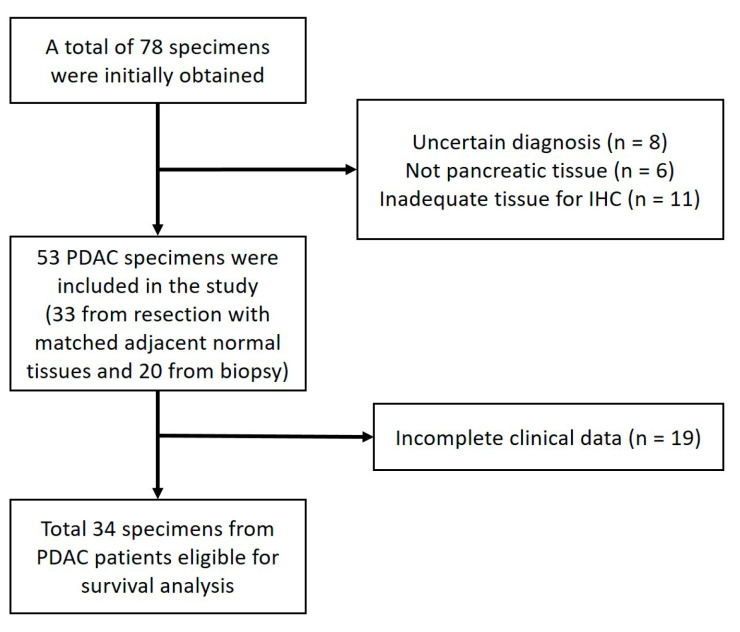
Flow diagram of patient recruitment. The flow diagram shows the number of patients and the specimens eligible for each stage of the study and the reason for any exclusions. PDAC, pancreatic ductal adenocarcinoma; and IHC, immunohistochemistry.

Figure 1 shows the flow diagram of the patient recruitment process from five hospitals and the number of eligible PDAC specimens in this study.

The inclusion criteria included the following: patients with PDAC who had undergone a resection or biopsy from January 2014 to December 2019. The clinical data were obtained from medical records. Patients were excluded if their relevant clinicopathologic data could not be obtained from these medical records. 

Our study was an exploratory study. Therefore, we attempted to include all the available PDAC specimens from the participating hospitals, and we managed to obtain 53 formalin−fixed, paraffin−embedded tissues from the eligible PDAC patients. Of those, 33 of the tissues were obtained from resections, and 20 were obtained from biopsies. The histologic sections were re−examined by two pathologists from Cipto Mangunkusumo National General Hospital to confirm the diagnosis of PDAC. We obtained the patients’ relevant clinicopathological data from their medical records. The extracted variables included the patients’ gender, age, tumor grade, the presence of neural invasion, the presence of lymphovascular invasion, the cancer stage, and the chemotherapy status. We also obtained the survival status of the patients from the medical records, or by phone if this was not available in the medical record (the last follow−up took place on 31 March 2020). The overall survival (OS) was defined as the time from the procedure to the date of death or the last follow−up. The patients who were still alive at the last follow−up were censored during the survival analysis. The median follow−up time was 38.5 months (reverse Kaplan–Meier method).

### 2.5. Immunohistochemistry

Xylol was used for the deparaffinization of the tissues, followed by a sequential rehydration with alcohol. We performed an antigen retrieval by heating the samples with TRIS EDTA for 20 min. Novocastra™ Peroxidase Block, followed by an additional protein block, was then applied to the tissues to decrease the non−specific staining. For the immunostaining, we used the following primary antibodies: NF−κB p65 (Cell Signaling Technology Cat# 8801, RRID:AB_2797670), anti−COX−2−antibody (Cell Signaling Technology Cat# 13314, RRID:AB_2798178), SP1 (Cell Signaling Technology Cat# 74315, RRID:AB_2799855), and c−Jun (Cell Signaling Technology Cat# 40502, RRID:AB_2909794). Next, the slides were incubated in a moist chamber for one hour. After washing them with a poly−buffered saline, we applied the post−primary antibody (Rabbit anti−Mouse IgG) to the slides. Next, we applied the Novolink™ Polymer (Anti−rabbit Poly−HRP−IgG). Then, diaminobenzidine (DAB) solution was added, and a counterstain was carried out with hematoxylin–eosin and lithium to produce a blue background. The slides were then dehydrated with an increasing concentration of ethanol, followed by a xylol application. 

### 2.6. Evaluation of Staining

Each slide was evaluated independently by two trained researchers that were blinded to the patients’ clinical data, in order to minimize bias. For each tumor lesion, the researchers assessed at least five representative high−power fields (400×). We used an Olympus BX50 light microscope. For COX−2, we evaluated only the cytoplasmic stain, since it was the dominant stain in our samples. For Sp1 and c−Jun, we only assessed the nuclear stain as the predominant stain. For NF−κB RelA/p65, we evaluated both the cytoplasmic and nuclear stains, since previous studies had assessed both [25]. We also evaluated the corresponding, matched, and normal adjacent tissues in the 33 available specimens, in order to compare the protein expressions in these tissues with the cancerous tissues.

We used two different positivity criteria for the nuclear and cytoplasmic staining. We considered the nuclear staining to be positive when the average number of cells in which the nucleus was stained was ≥50%. For the cytoplasmic staining, we calculated the expression scores based on the percentage of cells that were stained and the intensity of the stain. Based on the percentage of the stained cells, the scores were 0 (0%), 1 (1–25%), 2 (26–50%), 3 (51–75%), and 4 (76–100%). Based on the intensity of the stains, the scores were 0 (negative), 1 (weak), 2 (moderate), and 3 (strong). We then calculated the total score from these two domains (range of scores: 0–7). The cytoplasmic staining was categorized as positive if the total score was >3.5. 

### 2.7. Data Analysis

We present the continuous data as the mean and standard deviation, in order to describe the baseline characteristics. We present the categorical data as numbers and percentages. The missing data were managed via case deletion for that particular variable. We used Spearman’s rank test to analyze the correlation between COX−2 and its transcription factors (NF−κB RelA/p65 (cytoplasmic or nuclear expressions), Sp1, and c−Jun) in the cancerous tissues. We used the Mann–Whitney U test to evaluate the differences between NF−κB RelA/p65 (cytoplasmic or nuclear expressions), COX−2, Sp1, c−Jun, and the co−expressions of these four proteins in the cancerous tissues, and those in the adjacent normal tissues from 33 patients. In addition, we analyzed the difference between the ratio of the nuclear to cytoplasmic NF−κB RelA/p65 expressions between the cancerous and adjacent normal tissues. This ratio was calculated by dividing the percentage of the cells with positive nuclear staining (range 0–100%) by the total score of the cytoplasmic staining (0–7). The Wilcoxon signed−rank test was used to determine the differences in the ratio between the cancerous tissues and normal tissues. We also used the chi−square or Fisher’s test to investigate the association between the expression of the four proteins and the other clinicopathological data. 

For the survival analysis, we created Kaplan–Meier curves based on the status of the NF−κB RelA/p65 (cytoplasmic and nuclear), COX−2, Sp1, and c−Jun expressions. Next, we performed a univariate Cox regression analysis to calculate the hazard ratio (HR), and a multivariate COX regression analysis (backward stepwise LR method) to calculate the adjusted hazard ratio (aHR) based on the NF−κB RelA/p65 (cytoplasmic and nuclear), COX−2, Sp1, and c−Jun expression statuses. The other relevant clinicopathological data (gender, age, tumor grade, the presence of perineural or lymphovascular invasion, the type of procedure, and the tumor stage) were also included in the model. We also performed a separate Cox regression analysis for the patients with a known chemotherapy status. *p* ≤ 0.05 was considered to be statistically significant. We used IBM SPSS Statistics for Windows, v20, (RRID:SCR_016479) for the statistical analysis.

## 3. Results

### 3.1. Clinicopathological Characteristics

Table 1 shows the clinicopathological parameters of the patients. The median age of the patients was 51 (30–77) years old. More than 70% of the tumors were grade I or II. Most of the specimens did not show a perineural or lymphovascular invasion. 

### 3.2. NF−κB, COX−2, Sp1, and c−Jun Expressions in PDAC Tissues

**Figure 2:**
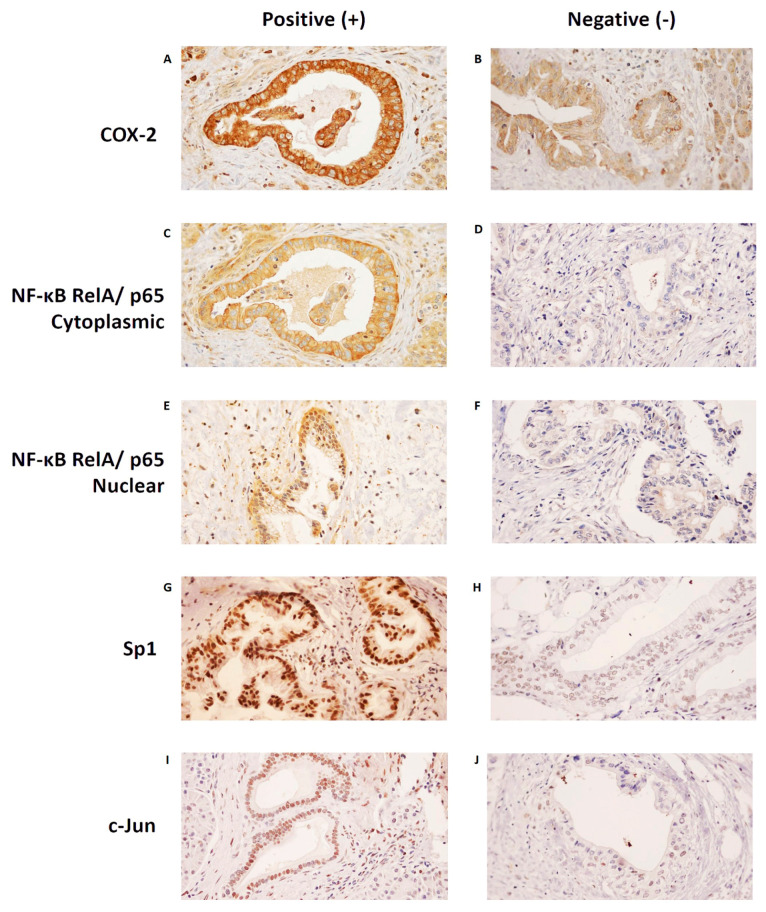
Positive and negative immunohistochemistry staining patterns of each protein. The most dominant stain of COX−2 in our specimens was in the cytoplasm. (**A**) An example of positive COX−2 cytoplasmic staining, and (**B**) an example of negative COX−2 cytoplasmic staining. The most dominant stain of NF−κB RelA/p65 in our specimens was also in the cytoplasm, although nuclear staining could also be seen. (**C**) An example of positive NF−κB RelA/p65 cytoplasmic staining, and (**D**) an example of negative NF−κB RelA/p65 cytoplasmic staining. (**E**) An example of positive NF−κB RelA/p65 nuclear staining, and (**F**) an example of negative NF−κB RelA/p65 nuclear staining. The most dominant stains of Sp1 and c−Jun in our specimens were in the nucleus. (**G**) An example of positive Sp1 nuclear staining, and (**H**) an example of negative Sp1 nuclear staining. (**I**) An example of positive c−Jun nuclear staining, and (**J**) an example of negative c−Jun nuclear staining. Images were taken at 400× magnification. COX−2, Cyclooxygenase−2; NF−κB, Nuclear Factor Kappa−B; Sp1, Specificity Protein 1.

Figure 2A–J shows examples of each protein’s positive and negative stains. For COX−2 and NF−κB, the dominant stains in our specimens were those for the cytoplasms of the ductal cells. For Sp1 and c−Jun, the dominant stains were those for the nucleus of the ductal cells.

### 3.3. Correlations between NF−κB, COX−2, Sp1, and c−Jun Expressions in PDAC Tissues

**Figure 3:**
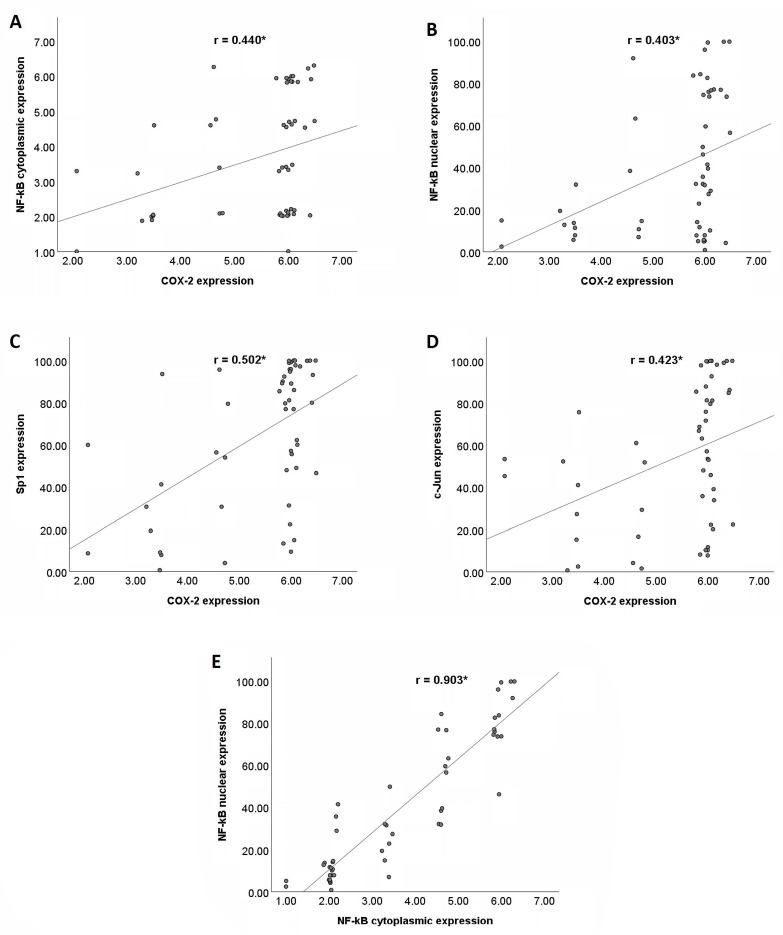
Correlations between COX−2 and its transcription factors (NF−κB, Sp1, and c−Jun) in PDAC tissues. (**A**) Positive correlation between COX−2 and cytoplasmic NF−κB expression. (**B**) Positive correlation between COX−2 and nuclear NF−κB expression. (**C**) Positive correlation between COX−2 and Sp1 expression. (**D**) Positive correlation between COX−2 and c−Jun expression. (**E**) Positive correlation between cytoplasmic and nuclear NF−κB expression. COX−2, cyclooxygenase−2; NF−κB, nuclear factor kappa−B; and Sp1, specificity protein 1. * *p* < 0.01.

Figure 3A–D shows the correlations between COX−2 and the NF−κB (cytoplasmic and nuclear), Sp1, and c−Jun expressions in the PDAC tissues. There were statistically significant positive correlations between COX−2 and the three transcription factors (NF−κB, Sp1, and c−Jun). The strengths of these correlations were fair to moderate. However, Figure 3E shows that there were very strong positive correlations between the cytoplasmic and nuclear NF−κB expressions.

### 3.4. Difference in NF−κB, COX−2, Sp1, and c−Jun Expressions between PDAC and Normal Tissues

**Figure 4:**
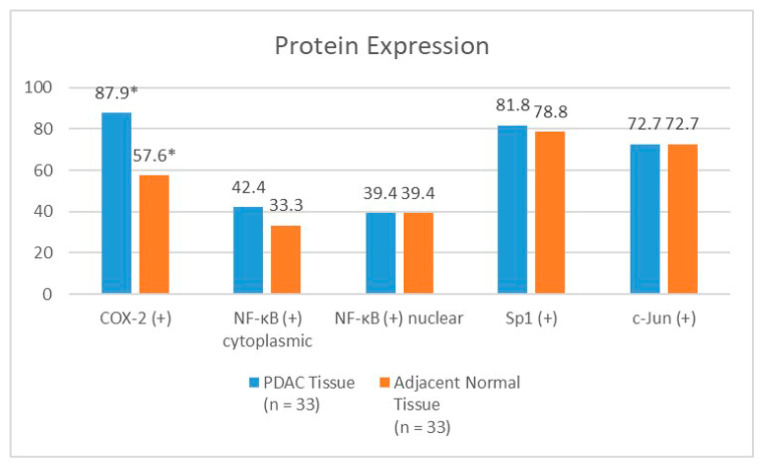
The difference in NF−κB, COX−2, Sp1, and c−Jun expressions between PDAC tissue and adjacent normal tissue. COX−2, cyclooxygenase−2; NF−κB, nuclear factor kappa−B; and Sp1, specificity protein 1. * *p* < 0.05.

Figure 4 shows the difference in the percentages of the cells expressing NF−κB (cytoplasmic and nuclear), COX−2, Sp1, and c−Jun between the PDAC and adjacent normal tissues. We were able to evaluate the adjacent normal tissues from 33 specimens. The PDAC tissues showed a higher COX−2 positivity than the adjacent normal tissues, with a statistically significant difference. There were no significant differences in the NF−κB (cytoplasmic and nuclear), Sp1, or c−Jun expressions between the PDAC and adjacent normal tissues. The ratio of the cytoplasmic to nuclear NF−κB expression was slightly higher in the cancerous tissues (median 11.95) than in the adjacent normal tissues (median 7.88), although this was not statistically significant (*p* = 0.357). There were no significant differences (*p* = 0.648) in the co−expressions of any of the four proteins (COX−2, nuclear NF−κB, Sp1, and c−Jun) between the PDAC tissues (24.5%) and the adjacent normal tissues (18.9%) either.

### 3.5. Associations between NF−κB, COX−2, Sp1, and c−Jun Expressions and the Clinicopathological Characteristics of PDAC Patients

Table S1 shows the percentage of the specimens that expressed NF−κB, COX−2, Sp1, and c−Jun, and their associations with the various clinicopathological characteristics. In total, thirty−four patients with complete medical records were included in this analysis. None of the clinicopathological factors had a significant association with either the NF−κB or COX−2 positivity. The specimens that were obtained from the resections had higher Sp1 and c−Jun positivities than those that were obtained from the biopsies.

### 3.6. Survival Curves

**Figure 5:**
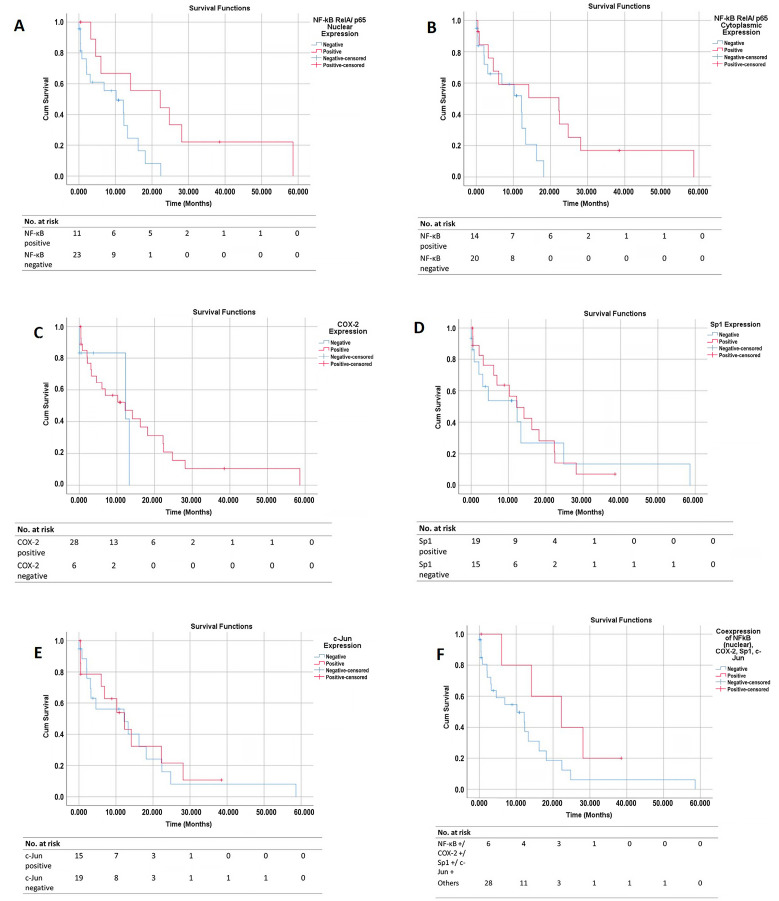
Overall survival (OS) curves based on the protein expression in PDAC tissues. The graphs show the OS curves based on (**A**) NF−κB (RelA) nuclear expression, (**B**) NF−κB (RelA) cytoplasmic expression, (**C**) COX−2 expression, (**D**) Sp1 expression, and (**E**) c−Jun expression. (**F**) OS curve of patients with co−expressions of all four proteins vs. other patient groups. OS, overall survival; COX−2, Cyclooxygenase−2; NF−κB, Nuclear Factor Kappa−B; and Sp1, Specificity Protein 1.

A total of twenty−four patients (70.6%) reached the outcome event (mortality) at the end of the follow−up period. Figure 5A–E shows the overall survival (OS) curves of the PDAC patients based on their NF−κB cytoplasmic, NF−κB nuclear, COX−2, Sp1, and c−Jun tissue expressions, respectively. Figure 5F shows the overall survival (OS) curves of the PDAC patients based on the tissue co−expressions of all four proteins.

The patients with a positive NF−κB cytoplasmic expression had a better OS than those with a negative expression (a median survival of 22.3 months vs. 12.2 months, *p* = 0.050). The patients with a positive NF−κB nuclear expression had a better prognosis than those with a negative expression (a median survival of 22.3 months vs. 10.2 months; *p* = 0.016). The survival curve that was based on the COX−2 status showed that, from the beginning to the 13th month, the patients with a positive COX−2 expression had a trend of worse OS than those without COX−2 expression. From the 13th month onwards, the opposite pattern was seen; the patients with a positive COX−2 expression had the trend of a better prognosis than those without COX−2 expression. Overall, the log−rank test showed no significant difference in the OS between the patients with or without COX−2 expression (a median survival of 12.2 months vs. 12.3 months; *p* = 0.528). A time−dependent Cox regression did not show any significant association between the COX−2 expression and patient survival either. 

The survival curve that was based on the Sp1 expression showed that, from the beginning to the 22nd month, the patients with a positive Sp1 expression had the trend of a better OS than those without Sp1 expression. However, from the 22nd month onwards, the patients with a positive Sp1 expression showed the trend of a poorer OS than those without Sp1 expression. Overall, the log−rank test showed no significant difference in the OS between those with and without Sp1 expression (a median survival of 12.2 months vs. 12.3 months; *p* = 0.830). A time−dependent Cox regression did not show any significant association between the Sp1 expression and patient survival either. The survival curve that was based on the c−Jun expression showed a similar OS for the patients with and without c−Jun expression (12.3 months vs. 12.2 months; *p* = 0.799). The patients with co−expressions of all four proteins (NF−κB nuclear +/COX−2 +/Sp1 +/c−Jun +) had the trend of a better OS than the other patient groups, although this was statistically insignificant (a median survival of 8.9 months vs. 5.0 months; *p* = 0.123).

### 3.7. Cox Regression Analysis

Table 2 shows the univariate Cox regression analysis results for all the predictors. From the univariate analysis, only the positive NF−κB nuclear expression reached statistical significance.

We also performed a multivariate Cox regression analysis (backward stepwise LR method) that was based on two models of the predictors, in order to assess the effect of the NF−κB cytoplasmic and nuclear expressions separately. The first model included the NF−κB cytoplasmic expression, the other three proteins (COX−2, Sp1, c−Jun), and the other clinicopathological predictors. In the first model, only the positive NF−κB cytoplasmic expression was independently associated with a longer OS (aHR = 0.31; 95% CI 0.11–0.90; *p* = 0.032). Meanwhile, the second prognostic model included the NF−κB nuclear expression, the other three proteins, and the other clinicopathological predictors. From the second model, the positive NF−κB nuclear expression was independently associated with a better prognosis (aHR = 0.22; 95% CI 0.07–0.66; and *p* = 0.007). In addition, the positive c−Jun expression reached statistical significance for its independent association with a poorer OS (aHR = 4.01; 95% CI 1.13–14.27; and *p* = 0.032). The specimens that were obtained from biopsies were also independently associated with a poorer OS (aHR = 3.69; 95% CI 1.12–12.20; and *p* = 0.032). No other factor was independently associated with survival. 

We also conducted a Cox regression analysis separately for the patients who had completed chemotherapy, as shown in their medical records (n = 27). Around 60% of the patients had received chemotherapy. Their chemotherapy regimens included gemcitabine, capecitabine, or 5−fluorouracil/folinic acid. The Cox regression analysis, including the protein expressions, chemotherapy status, and other clinicopathological data, showed that the nuclear NF−κB expression was still independently associated with a better prognosis in the PDAC patients (aHR = 0.16; 95% CI 0.02–0.98; and *p* = 0.048). The male sex (aHR = 12.91; 95% CI 1.34–124.35; and *p* = 0.027), a younger age (<60 years old) at diagnosis (aHR = 46.01; 95% CI 3.01–703.83; and *p* = 0.006), and having not undergone chemotherapy (aHR = 37.46; 95% CI 2.51–558.14; and *p* = 0.009) were independently associated with a worse prognosis. In a separate model, the cytoplasmic NF−κB expression was associated with a better prognosis in the PDAC patients, although this was not statistically significant (aHR = 0.40; 95% CI 0.10–1.68; and *p* = 0.214). The other variables showed no significant associations with the prognosis.

## 4. Discussion

To our knowledge, our study is the first to show that there are significant correlations between the NF−κB, COX−2, Sp1, and c−Jun in PDAC tissues via immunohistochemistry. The promoter region of the *COX2* gene contains binding sites for various transcription factors, including NF−κB, Sp1, and c−Jun. However, the relative contribution of each transcription factor to the regulation of the *COX2* gene expression depends on the specific stimulus and the types of cells [11]. Our findings show that NF−κB, Sp1, and c−Jun had significant correlations with the COX−2 expression, which suggests that all three proteins have significant roles in regulating the COX−2 expression in PDAC cells. This finding is corroborated by previous studies that have investigated the correlation between Sp1 and COX−2 in PDAC patients’ tissues [26,27]. Hang J et al. reported a significant moderate correlation between the Sp1 and COX−2 in PDAC tissues (r = 0.599, *p* < 0.001) [26]. Likewise, another study by Hu et al. demonstrated a positive correlation between the Sp1 and COX−2 in PDAC cells (r = 0.353, *p* < 0.001) [27].

Our present study also showed that the rate of the positive COX−2 expression was higher in the cancerous tissues than in the adjacent normal tissues. This result is consistent with previous studies [9,10]. Yip−Schneider et al. used the immunoblot technique to determine the difference in the rate of the COX−2 expression between 23 PDAC tissues and 11 adjacent, matched, normal tissues. Densitometry demonstrated that the median percentage of the COX−2 expression in the pancreatic cancer tissues was significantly higher than that in the adjacent normal tissues (5.2% vs. 0.2%) [10]. Maitra A et al. compared the COX−2 expression between PDAC, pancreatic intraepithelial neoplasia (PanIN), and normal pancreatic duct cells. They used an aggregate scoring system based on the percentage of the cells that were stained and the intensity of the staining (HistoScore), which was similar to our scoring system. Their results showed significantly higher scores in the PDAC tissues than in the normal tissues. In addition, there was a trend of a higher score in the PDAC tissues compared with the PanIN tissues [9]. 

Only one study has investigated the difference in the NF−κB expression between the PDAC and normal tissues in PDAC patients using immunohistochemistry. Zhang JJ et al. obtained 65 specimens from PDAC patients, and 38 matched normal tissues. Their results showed that the rate of the positive NF−κB expression was higher in the PDAC tissues compared with normal tissues (66.5% vs. 31.58%) [14]. Likewise, few studies have analyzed the difference in the Sp1 or c−Jun expression between PDAC and normal tissues. Hu J et al. reported that positive Sp1 expression was higher in the PDAC tissues than in the normal tissues, although they did not report the exact percentage [16]. Ferrara C et al. reported that the percentage of the cells that were stained with c−Jun in the PDAC tissues ranged from 10 to 90%, while only 10% of the ductal cells were stained in the normal tissues [18]. Our present study showed no significant differences in the NF−κB, Sp1, and c−Jun expressions between the PDAC tissues and the adjacent normal tissues. This result might have been caused by our study’s relatively low number of patients. In addition, there might have also been background inflammation still present in the normal adjacent tissues. The additional inflammatory markers in the adjacent normal tissues should be evaluated in future studies. We also found that the ratio of nuclear to cytoplasmic NF−κB expression was higher in the PDAC tissues than the adjacent normal tissues, which might reflect higher rates of NF−κB activation, although this was not statistically significant. In addition, we found that the Sp1 and c−Jun expressions were higher in the specimens that were obtained from resections than those that were obtained from biopsies. This association might be caused by the greater amount of tissue mass that could be analyzed in the specimens that were obtained from resections than those from biopsies. The higher amount of tissue mass in the resection specimens might provide a more representative inflammatory profile.

The most surprising finding in the present study was that a positive NF−κB expression, either in the cytoplasm or the nucleus of malignant ductal cells, was associated with a better prognosis in the PDAC patients. In total, two previous studies have investigated the association of NF−κB expression with the survival of PDAC patients via immunohistochemistry. Weichert W et al. reported that a positive cytoplasmic and nuclear NF−κB expression was associated with a worse OS than negative expressions. However, in the Cox regression analysis, the NF−κB expression in the cytoplasm (*p* = 0.235) or nucleus (*p* = 0.120) was not independently associated with the patient’s survival. In a subgroup analysis of the patients with a node−negative status, the cytoplasmic NF−κB expression was independently associated with a poor prognosis (RR = 3.49; *p* = 0.020) [25]. A study by Yang SH et al. demonstrated that patients with a positive nuclear NF−κB expression had a poorer OS than those with a negative or only cytoplasmic expression (a median survival of 5.5 months vs. 13.9 months; *p* < 0.001). They also showed that patients with a positive nuclear NF−κB expression had a worse OS post−chemotherapy than the other patient groups (the median survival after chemotherapy was 3.0 months vs. 7.0 months; *p* < 0.001). This association holds when controlling for other variables (*p* = 0.02) [28]. Overall, the pooled results of these two studies from our meta−analysis showed that patients with a positive NF−κB expression had a worse OS than those without it, but this was not significant when controlling for other variables (aHR = 2.38; 95% CI 0.68–8.25) [24]. 

Although the c−Jun expression was not significantly associated with patient survival in the univariate analysis, it was found to be significant in the multivariate analysis. In one of the Cox regression models, the c−Jun expression was associated with a worse prognosis in the PDAC patients. To our knowledge, only one study had previously investigated this association between c−Jun expression and PDAC patient survival via immunohistochemistry. Ferrara C et al. reported that patients with a higher c−Jun expression were associated with a shorter OS (*p* = 0.03), although this was not significant when controlling for other variables [18]. Our study did not reveal a significant association between COX−2 expression and patient survival, although there was a trend of a worse OS in the first 13 months for patients with COX−2 expression than for those without. This suggests that COX−2 increases the severity of the disease in the early stages of cancer, but not in the late stages. Previous studies that have investigated the prognostic significance of COX−2 in PDAC showed inconsistent results. A study by Fagman et al. on 32 patients with PDAC showed that COX−2 expression was not independently associated with survival [19]. Meanwhile, several studies have reported that COX−2 expression was independently associated with a worse OS for PDAC patients [20,21,26]. Juuti et al. showed that a positive COX−2 expression was independently associated with a worse prognosis in PDAC patients (aHR = 1.6; 95% CI 1.1–2.4) [20]. In contrast, a study by Pomianowska et al. on 92 patients with PDAC demonstrated that patients with a positive COX−2 expression had a better OS than those without one (a median survival of 18 months vs. 11 months). Positive COX−2 expression was also independently associated with a better prognosis in that cohort of patients (aHR = 1.64; 95% CI 1.01–2.68). One explanation that they proposed was that tumors with positive COX−2 expressions have lower histological grades than those with negative expressions [22]. Our previously published meta−analysis showed that, overall, PDAC patients with a positive COX−2 expression had trends of a lower OS than those with negative expressions. However, that association was insignificant when the other clinicopathological variables were controlled (aHR = 1.30; 95% CI 0.80–2.13) [24]. 

We did not find a significant association between Sp1 expression and patient survival either, although a slightly worse OS from the 22nd month onwards was observed. Previous studies have shown that a positive Sp1 expression in PDAC, as assessed via immunohistochemistry, was associated with a worse survival in its patients. Hang J et al. studied the association of Sp1 and COX−2 expression with the overall survival in 88 PDAC patients. They reported that patients with a positive Sp1 or COX−2 expression were independently associated with a worse prognosis [26]. Jiang et al. showed that Sp1 expression was associated with a higher tumor grade, a higher rate of lymph node metastasis, and a shorter OS [29]. Overall, our meta−analysis of previous studies showed that Sp1 expression is an independent predictor of a worse prognosis in PDAC patients (aHR = 3.47; 95% CI 1.52–7.94) [24]. We also found that the co−expression of all four proteins (nuclear NF−κB +/COX−2 +/Sp1 +/c−Jun +) was associated with a longer OS than that in other patient groups, although this was statistically insignificant. No previous study has analyzed the co−expression of these four proteins. Hang J et al. did show that PDAC patients with a co−expression of Sp1 and COX−2 had a shorter OS than the other patient groups in a univariate analysis. However, this lost its statistical significance in the multivariate analysis [26].

Overall, our findings suggest that the involvement of transcription factors and inflammatory mediators in PDAC might be more complex than previously thought. Most previous studies have stated that these transcription factors (NF−κB, Sp1, and c−Jun) and COX−2 play a role in sustaining oncogenic activity, and thus would also be associated with a poor prognosis in PDAC. For example, Hill R et al. discovered that pancreatic cancer cells’ intrinsic COX enzyme could upregulate the *p*−AKT levels, leading to an increased cell proliferation [30]. The level of COX−2 mRNA also showed a significant positive correlation with matrix metalloproteinase−9 (MMP−9), which plays a role in cancer metastasis [31]. The PGE2 that was synthesized from COX−2 was also associated with an increased cellular proliferation, anti−apoptosis, cellular migration, and angiogenesis [32]. NF−κB can be activated by the classical or alternative pathway, which results in its translocation from the cytoplasm to the nucleus, allowing it to induce the expression of target genes. By expressing target genes such as *COX2*, NF−κB can exert a pro−tumor effect and chemotherapeutic resistance in patients with PDAC [13]. In turn, COX−2 can increase the NF−κB activity by producing prostaglandins [33]. Sp1 expression can lead to the upregulation of several target genes, including COX−2. This leads to an increased VEGF secretion, which promotes angiogenesis [27]. Increased AP−1 binding activity, which was conferred by c−Jun, was observed in pancreatic cancer cells. This increased binding activity was associated with increased cellular proliferation [34].

However, NF−κB, COX−2, Sp1, and c−Jun can become double−edged swords in carcinogenesis. Although more seldomly discussed, these four proteins can have anti−tumor properties in a certain context. After all, inflammation can also be beneficial in suppressing cancer, as shown by the application of immunotherapy in various cancers, including in PDAC patients with a mismatched repair deficiency (dMMR) [35]. NF−κB can exert anti−tumor properties by promoting cellular death through the suppression of anti−apoptotic genes, synergizing with tumor suppressors such as p53, reducing cellular proliferation by inhibiting JNK, and resolving inflammation in later stages [36]. Several in vitro and animal studies have shown that NF−κB activation could lead to tumorigenesis [37,38]. In terms of human studies, patients with gastric cancer who expressed nuclear NF−κB in their cancer cells had a better OS than those with a negative or only cytoplasmic NF−κB expression (*p* = 0.0228). In addition, nuclear NF−κB expression was associated with earlier cancer stages and a less extensive lymphatic invasion [39]. These anti−tumor properties could explain why NF−κB expression was associated with a better PDAC prognosis in our study.

Likewise, COX−2 can have anti−tumor properties depending on the type of prostaglandin that is synthesized. Although PGE2 is the main eicosanoid that is linked with carcinogenesis, COX−2 can also produce PGD2, which has tumor suppressor activity [40]. This phenomenon could partly explain why the clinical trials that have investigated the addition of COX−2 inhibitors to the standard chemotherapy for PDAC patients did not observe improved patient outcomes [41,42]. Using various cancer cell lines, Chuang JY et al. also showed that an Sp1 overexpression could induce apoptosis and suppress the cell growth in transforming cells. However, these effects were also dependent on a functional p53 protein [43]. Although c−Jun has been linked to tumorigenesis, other members of the JUN family of AP−1 (such as JunB and JunD) were associated with anti−tumor activities instead [17].

In this study, we only investigated the expressions of the four proteins in the ductal cells. However, the tumor microenvironment (TME) also plays a significant role in the pathophysiology of PDAC. The TME in PDAC consists of cytokines, metabolites, cancer−associated fibroblasts, and desmoplastic stroma, which helps the tumor cells to evade the host’s immune system. The TME also comprises infiltrating CD8+ T cells, tumor−associated macrophages, and myeloid−derived suppressor cells [3]. These cells could be a source of inflammatory molecules. Omura N et al. showed that some pancreatic cancer cells from cell lines do not express the *COX1* or *COX2* genes. However, these cells could use the prostaglandin that is produced by the exogenous COX enzymes, such as that from fibroblasts. When performing a knockdown of the prostaglandin transporter in fibroblasts, they discovered a reduction in the cancer cell proliferation. These findings indicate that COX−deficient pancreatic cancer cells can still use the exogenous COX enzymes from the cells in the tumor microenvironment [44]. This mechanism might partly explain the wide range of the positive COX−2 expressions in PDAC cells that have been reported in previous studies [20,22]. Treiber M et al. demonstrated that NF−κB expression produces different effects in different types of cells. Using chronic pancreatitis mouse models, they showed that the NF−κB RelA/p65 expressions in acinar cells were associated with protective effects against inflammation. In contrast, the NF−κB RelA/p65 expressions in myeloid cells promoted fibrogenesis by activating pancreatic stellate cells (PSCs) [45]. This could explain why the NF−κB expression in the cancerous ductal cells in our study was associated with a better prognosis in the PDAC patients. 

The limitations of this study include the small number of samples that were analyzed, even though we collected the samples from multiple centers and included specimens from biopsies. This low number of samples might reflect either a low incidence or underdiagnosis. It was also difficult to obtain complete medical records, which reflected the need for a national pancreatic cancer registry. Despite this, our results show a clear pattern of the association between NF−κB expression and a better prognosis in PDAC patients, independent of other clinicopathological factors. 

In conclusion, our results suggest that the role of inflammation in PDAC is more complex than it first seems. Due to their possible opposing effects in carcinogenesis, it could be challenging to use the expressions of NF−κB, COX−2, Sp1, and c−Jun in ductal cells, as assessed via immunohistochemistry, as the prognostic factors in PDAC patients. Further research is needed to identify the factors that might influence the pro−tumor or anti−tumor effects of each of these four proteins. It is noteworthy that the patients that were included in our study were relatively younger (a median age of 52 years old) compared to the reports from the USA (a median age of 70 years old) [46]. Approximately 41.8% of our patients had early onset pancreatic cancer, which was defined as PDAC diagnosed before 50 years old [47]. This finding is similar to another report from a hospital in Indonesia, wherein the median age of the PDAC patients was 53.8 years old [48]. It is unclear whether patient age is associated with these four proteins’ expressions, although no association was noted in our study. Further studies are needed to delineate the potential covariates that might affect these protein expressions and patient survival. Another alternative strategy for studying these potential prognostic factors in PDAC would be to investigate the expression of the other subunits of NF−κB (such as RelB, c−Rel, NF−κB1, or NF−κB2) and AP−1 (proteins from the FOS family or other members of the JUN family), or the downstream targets (such as the prostaglandins in COX−2). Several studies have reported that the higher urinary levels of PGE2 were independently associated with an increased risk of PDAC [49,50]. Another important area of study is the use of inflammatory markers such as NF−κB to predict the response of immunotherapy in PDAC. This is because NF−κB can also regulate the expression of PD−L1 in cancers [51]. 

## Figures and Tables

**Table 1:** Clinicopathological characteristics.

Characteristics	Categories	Number (%)
Mean age in years (standard deviation)		52.4 (SD 10.9)
Gender	Male	33 (62.3)
	Female	20 (37.7)
Nuclear grade	I	22 (41.5)
	II	16 (30.2)
	III	15 (28.3)
Lymphovascular invasion	Yes	6 (11.3)
	No	47 (88.7)
Perineural invasion	Yes	15 (28.3)
	No	38 (71.7)
Specimen	Resection	33 (62.3)
	Biopsy	20 (37.7)

SD = standard deviation.

**Table 2:** Results of univariate analysis.

Factors	Total	Univariate Analysis		
		HR	95% CI	*p*−Value
Gender				
Male	19	1.72	0.74–4.03	0.210
Female	15	1		
Age				
≥60	11	0.85	0.35–2.09	0.731
<60	23	1		
Grade				
>1	20	1.55	0.66–3.65	0.311
1	14	1		
Perineural invasion				
Present	11	1.02	0.42–2.50	0.959
Not Present	23	1		
Lymphovascular invasion				
Present	6	0.50	0.15–1.70	0.268
Not Present	28	1		
Type of specimen				
Biopsy	13	1.49	0.65–3.41	0.341
Resection	21	1		
Cancer stage				
III–IV	24	1.82	0.60–5.55	0.289
I–II	10	1		
NF−κB (RelA) cytoplasmic status				
Positive	14	0.37	0.14–1.03	0.058
Negative	20	1		
NF−κB (RelA) nuclear status				
Positive	11	0.29	0.10–0.84	0.022 *
Negative	23	1		
COX−2 status				
Positive	28	0.67	0.19–2.35	0.531
Negative	6	1		
Sp1 status				
Positive	19	0.91	0.39–2.12	0.830
Negative	15	1		
c−Jun status				
Positive	15	0.90	0.39–2.06	0.799
Negative	19	1		
Co−expressions of NF−κB (nuclear), COX−2, Sp1, c−Jun				
Yes	6	0.43	0.14–1.29	0.133
No	28	1		
Gender				

Abbreviations: COX−2 = cyclooxygenase−2; NF−κB = nuclear factor kappa−B; Sp1 = specificity protein 1; HR = hazard ratio; and 95% CI = 95% confidence interval. * *p* < 0.05.

## Data Availability

Data are available upon request to the corresponding author.

## Supplementary Materials

The following supporting information can be downloaded at: https://www.mdpi.com/article/10.3390/pathophysiology30020009/s1. Table S1: Associations of NF−κB, COX−2, Sp1, and c−Jun tissue expressions with other clinicopathological characteristics in PDAC patients.
