# Supplementary material for: The Expressions of NF−κB, COX−2, Sp1, and c−Jun in Pancreatic Ductal Adenocarcinoma and Their Associations with Patient Survival
Source: Pathophysiology. 2023 Mar 25;30(2):92–109. doi: 10.3390/pathophysiology30020009 (PMC10123625; doi:10.3390/pathophysiology30020009)
Supplement: Supplementary file 1 [file pathophysiology-30-00009-s001.zip › pathophysiology-2132538-supplementary.pdf]

Table S1. Associations of NF-κB, COX-2, Sp1, and c-Jun tissue expressions with other clinicopathological characteristics in PDAC patients

Factors	Total	NF-κB (RelA) Cytoplasmic Positive (%)	*P*-value	NF-κB (RelA) Nuclear Positive (%)	P-value	COX-2 Positive (%)	*P*-value	Sp1 Positive (%)	*P*-value	c-Jun Positive (%)	*P*-value
**Gender**											
**Male**	19	7 (36.8)	0.563	5 (26.3)	0.475†	17 (89.5)	0.370†	9 (47.4)	0.260	6 (31.6)	0.097
**Female**	15	7 (46.7)		6 (40.0)		11 (73.3)		10 (66.7)		9 (60.0)	
**Age (years old)**											
**≥60**	11	5 (45.5)	1.000†	4 (36.4)	1.000†	9 (81.8)	1.000†	4 (36.4)	0.151†	5 (45.5)	1.000†
**<60**	23	9 (39.1)		7 (30.4)		19 (82.6)		15 (65.2)		10 (43.5)	
**Grade**											
**>1**	20	8 (40.0)	0.868	6 (30.0)	1.000†	17 (85.0)	0.672†	11 (55.0)	0.901	10 (50.0)	0.409
**1**	14	6 (42.9)		5 (35.7)		11 (78.6)		8 (57.1)		5 (35.7)	
**Perineural Invasion**											
**Present**	11	3 (27.3)	0.295†	3 (27.3)	1.000†	9 (81.8)	1.000†	7 (63.6)	0.715†	5 (45.5)	1.000†
**Not Present**	23	11 (47.8)		8 (34.8)		19 (82.6)		12 (52.2)		10 (43.5)	
**Lymphovascular Invasion**											
**Present**	6	4 (66.7)	0.202†	4 (66.7)	0.070†	6 (100.0)	0.562†	5 (83.3)	0.196†	4 (66.7)	0.370†
**Not Present**	28	10 (35.7)		7 (25.0)		22 (78.6)		14 (50.0)		11 (39.3)	
**Type of Specimens**											
**Biopsy**	13	7 (53.8)	0.238	4 (30.8)	1.000†	11 (84.6)	1.000†	4 (30.8)	0.020	2 (15.4)	0.008
**Resection**	21	7 (33.3)		7 (33.3)		17 (81.0)		15 (71.4)		13 (61.9)	
**Cancer Stage**											
**III-IV**	24	10 (41.7)	1.000†	7 (29.2)	0.692†	21 (87.5)	0.328†	13 (54.2)	1.000†	10 (41.7)	0.718†
**I-II**	10	4 (40.0)		4 (40.0)		7 (70.0)		6 (60.0)		5 (50.0)	
**Metastasis (stage IV)**											
**Present**	14	6 (42.9)	0.868	4 (28.6)	1.000†	12 (85.7)	1.000†	7 (50.0)	0.563	7 (50.0)	0.563
**Not Present**	20	8 (40.0)		7 (35.0)		16 (80.0)		12 (60.0)		8 (40.0)	
